# Toward Redox-Free Reverse Electrodialysis with Carbon-Based Slurry Electrodes

**DOI:** 10.1021/acs.iecr.2c03567

**Published:** 2023-01-14

**Authors:** Catarina Simões, Michel Saakes, Derk Brilman

**Affiliations:** † Wetsus, European Centre of Excellence for Sustainable Water Technology, PO Box 1113, Leeuwarden 8900 CC, The Netherlands; ‡ Sustainable Process Technology, Faculty of Science and Technology, University of Twente, PO Box 217, Enschede 7500 AE, The Netherlands

## Abstract

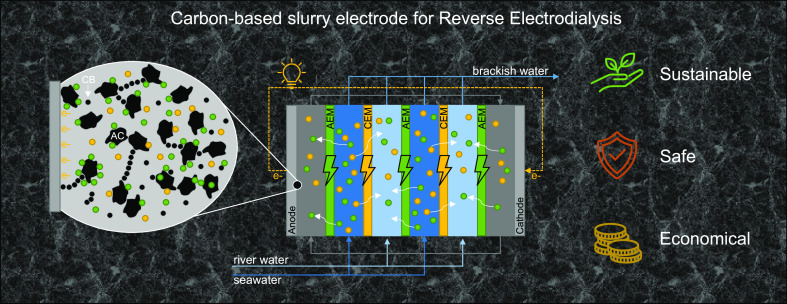

Clean and renewable salinity gradient energy can be harvested using reverse electrodialysis (RED). The electrode system is an essential part to convert ionic current into electrical current. In this study, a typical 0.10 × 0.10 m^2^ RED stack with a cross-flow configuration was used to test carbon-based slurry electrodes (CSEs) to replace the usual redox solutions, like hexacyanoferrate, to enhance the RED process’ sustainability, stability, and economic value. Six different slurry compositions comprising activated carbon, carbon black, and graphite powder were tested. The CSE characteristics were systematically studied by measuring viscosity, electrode compartment pressure drop, maximum current density, stability, and performance of power density and energy efficiency. Using a single membrane configuration, the CSE ran continuously for 17 days with a stable output. The application of CSEs for RED, with artificial seawater and river water, using mixing activated carbon and carbon black at a total concentration of 20 wt %, resulted in the best performance with a net power density of 0.7 W·m^–2^. Moreover, higher current densities up to 350 A·m^–2^ were tested for ED and shown to be feasible until 150 A·m^–2^. CSEs show promising versatility for different application modes.

## Introduction

Reverse electrodialysis (RED) is an emerging technology that generates renewable energy from the mixing of waters with different salinities, such as sea and river water.^1^ RED uses a membrane stack comprising alternating anion exchange membranes (AEMs) and cation exchange membranes (CEMs). Spacers separate the ion exchange membranes (IEMs) and, at the same time, shape the water compartments where seawater and river water are fed alternately.^2^ Due to the salinity gradient and the selective transport of anions and cations through the IEMs, an ionic current is produced at the membrane pile. Generally in RED, this ionic current is converted into an electrical current utilizing dimensionally stable electrodes at the end-compartments. Solutions, such as water, for electrolysis or redox couples, are used for electron transfer. The most used solutions for RED are the Fe^2+^/Fe^3+^ couple at low pH or hexacyanoferrate [Fe(CN)_6_]^3–^/[Fe(CN)_6_]^4–^ mixed with, e.g., 0.25 M NaCl or anthraquinone.^3^

Redox couples, like hexacyanoferrate, which is typically used at the laboratory scale, provide a fast charge transfer rate, and make the electrode system resistance negligible compared to the membrane pile resistance.^4^ However, their sustainability, stability, and economic viability are debatable for large-scale application. The Fe^2+^/Fe^3+^ couple is only stable at pH values below 2,^5^ requiring that the shielding membranes, positioned at the ends of the membrane pile, must be resistant to acidic environments. Furthermore, continuous pH monitoring combined with acid dosing is necessary to avoid the precipitation of iron compounds around the cathode.^3,4,6^ The [Fe(CN)_6_]^3–^/[Fe(CN)_6_]^4–^ couple decomposes in the presence of sunlight and oxygen. It was shown to be unstable under these circumstances and partially releases cyanide ions that, in the case of leakage, can irreversibly bind with the AEMs reducing their performance and harm the environment.^3,5,7^ Recently, this couple was also found to be unstable in scaled-up stacks in the presence of Mg^2+^ and Ca^2+^ where scaling occurred.^8^ In the case of electrolysis using NaCl solutions (or seawater), gas evolution occurs, and because of the fast kinetics of the chlorine evolution reaction gaseous chlorine gas (Cl_2_) evolves at the anode and hydrogen (H_2_) gas at the cathode. Chlorine gas is corrosive, while hydrogen gas increases the risk of explosion and must be removed from the system. Furthermore, gas bubbles at the electrode compartments will increase the electrical resistance leading to higher ohmic voltage losses.^9^ Therefore, there is a need for an alternative way to transfer electrons.

Static capacitive carbon electrodes have been developed for energy generation in RED (capacitive RED or CRED)^10^ and capacitive mixing (CAPMIX),^11^ as well as for desalination in electrodialysis (ED)^12^ and capacitive deionization (CDI).^13^ These are environmentally friendly since carbon is widely available and in case of leakage, there is no harm to the aquatic environment. The charge transfer mechanism is based on the ions being adsorbed onto the surface area of the carbon electrodes because of the electrostatic field of the electrical double layer,^14^ and no faradaic redox reactions occur. However, after adsorption saturation, it is necessary to reverse the polarity to trigger desorption, creating an intermittent charge and discharge process. Using CRED, an interruption in power generation is established as well as a mandatory feed water switch. The power density will be maximum at the start and decrease continuously during one cycle.^10^ This shows a limitation to the use of static electrodes, especially in cases where switching the process feed streams for discharge is not possible (e.g., asymmetrical compartments).

A possible solution without necessitating the periodic switch of river water and seawater is the use of carbon-based flow electrodes.^15^ The advantages of these flow electrodes compared to redox electrolytes are low cost, easy scalability, and being harmless to the ion exchange membranes and nonpolluting to the environment.^16^ Flow electrodes are widely used in flow capacitive deionization (FCDI) and electrochemical flow capacitors. In FCDI, it is used to continuously desalinate saline streams. By using a capacitive flow electrode, the process of adsorption and desorption can be made continuous and, in the case of desalination, leads to increased salt removal rates.^17^ In the case of capacitors, it is used as an energy storage device, by charging and discharging the flow electrode.^18^ Several studies have shown a continuous improvement of carbon flow electrodes by optimizing flow rates, preparation procedures, compositions, additives, and regeneration methods.^16,19−21^ In RED, the concept of a carbon flow electrode for reverse electrodialysis was first introduced by Liu et al.,^22^ where a RED stack was used with a capacitive carbon flow electrode at the end-compartments. Liu et al. experimented only with different activated carbon (AC) loads (5–15 wt %) and graphite brushes to enhance the contact area.^22^ However, the maximum power density achieved was rather low, namely, 0.29 W·m^–2^ using 1.0 and 30.0 g NaCl·L^–1^ solutions as feedwaters at a flow velocity of 1.0 cm·s^–1^.

Carbon-based flow electrodes usually contain carbon percentages lower than 25 wt %, to guarantee flowability in the long term.^23^ At higher carbon percentages, the flow electrode viscosity, and hence pressure drop, may become prohibitive, although with distinct designs the weight percentage of carbon may be increased.^24^ These flow electrodes are composed of micro- to nanosized particles, with AC being the main carbon material used.^17^ Although AC can offer high specific surface areas (∼1500–3200 m^2^·g^–1^), it has poor conductivity. Improvement of the flow electrode’s conductivity with additives was successful with the addition of, for example, carbon nanotubes^25^ and carbon black (CB),^26^ among others.^27,28^ Opposite to static capacitive electrodes, flow electrodes do not need a high capacitance since there is a continuous refreshment of the charged or discharged adsorption layer by neutralization of the flow electrode from both electrode compartments in a common mixing vessel or through recirculation from the anode to the cathode.^26^ Electrically conductive additives to AC, such as CB, can boost the electrical conductivity of the flow electrode and enhance the collision rate between the particles. CB with a low percolation threshold has been engineered to facilitate the charge transfer at a very low percolation threshold.^29,30^ To the best of our knowledge, a mixed slurry of AC and CB, or other electrically conductive additives, has not been demonstrated yet with carbon-based slurry electrodes in RED.

In this study, a typical 0.10 × 0.10 m^2^ RED stack with a cross-flow configuration was used to test carbon-based slurry electrodes (CSEs), as flow electrodes, to replace the usual redox solutions, like hexacyanoferrate, to enhance the RED process in terms of sustainability and stability. The CSE characteristics were systematically studied by measuring composition, viscosity, electrode compartment pressure drop, maximum current density, stability, and performance in terms of power density and energy efficiency.

One aim of this study was to study whether a CSE composed of a mix of different carbon particles has improved performance compared to flow electrodes using only AC, by testing using a standard RED stack. Another aim was to run a long-term test (17 days) to show the electrochemical stability of the best-performing CSE. Yet another aim was to evaluate the pressure drop of the CSE related to targeting a low pumping power consumption.

## Materials and Methods

### Carbon-Based Slurry Electrode Preparation and Characterization

CSEs were composed of AC, AC (YP-50F, Kuraray Corp., Japan), CB, (Monarch 800, Cabot, USA), and graphite powder (GP), (Graphite fine powder extra pure, Merck, USA) combined with deionized water and NaCl salt (VWR Chemicals, Belgium). The composition of each CSE can be found in Table 1. The salt concentration was fixed at 0.25 M NaCl having the average concentration of seawater and river water, to avoid osmosis. The total weight percentage of carbon was not more than 20%, to ensure flowability. The weight percentage of carbon (wt %) is the mass of carbon divided by the total mass of the slurry. Each CSE was prepared by individually weighing and then mixing all the elements first manually and after, for 12 min, with an UltraTurrax (IKA, T25, Germany) at 12,000 rpm. More details regarding the preparation can be found in the Supporting Information (Table S1). Later on, to improve the dispersibility of the CSE, a new CSE with 15 wt % AC, 5 wt % CB, and a surfactant, cetyltrimethylammonium bromide (CTAB, Sigma-Aldrich, USA), was prepared.

**Table 1:** Carbon-Based Slurry Electrode Composition Used in This Study

CSE #	name	AC [wt %]	CB [wt %]	GP [wt %]	NaCl [M]
1	20 AC	20	0	0	0.25
2	15AC5CB	15	5	0	0.25
3	10AC10CB	10	10	0	0.25
4	10AC5CB	10	5	0	0.25
5	10CB	0	10	0	0.25
6	15AC5GP	15	0	5	0.25

The AC, CB, and GP were characterized before making the CSEs. Samples were dried at 65 °C for 24 h prior to analysis, for scanning electron microscopy (SEM) to evaluate the surface morphology (JSM-6480LV, JEOL, Japan). All samples were degassed under a nitrogen atmosphere for 2 h at 300 °C in a degassing apparatus (VacPrep 061, Micromeritics, Norcross, GA, USA). Subsequently, nitrogen gas adsorption (TriStar 3000, Micromeritics, Norcross, GA, USA) at −196 °C was used to determine the specific surface area and pore size of the samples according to the Brunauer–Emmett–Teller (BET) analysis. From the slurries, samples were taken to determine the viscosity using a modular compact rheometer (MCR 102, Anton Paar, Austria) at shear rates from 1 to 400 s^–1^, at 22 °C. All slurry samples were shaken before being introduced into the rheometer to ensure homogeneity. The results of these characterizations can be found in the Supporting Information.

### Experimental Setup

**Figure 1:**
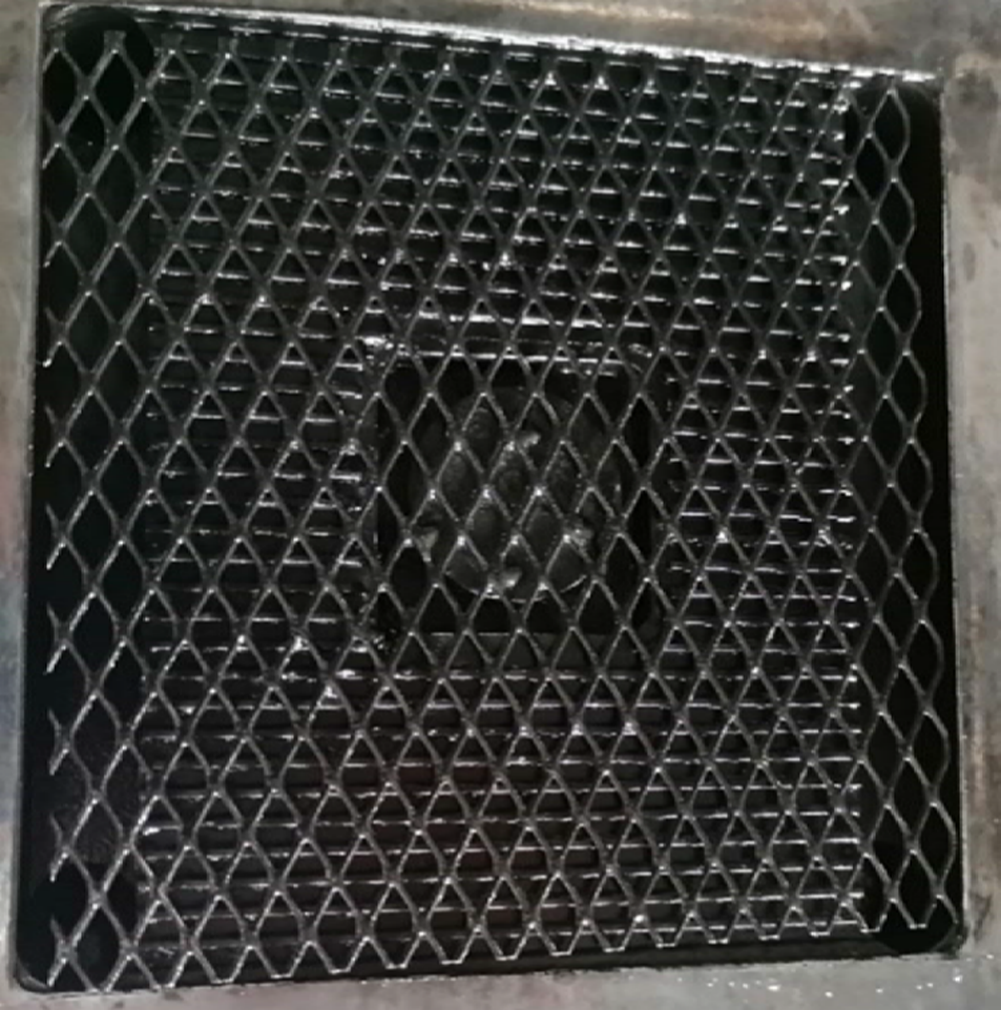
Flow geometry of the electrode compartment: Pt/Ti mesh on top, perpendicular flow field below, a manifold both for the inlet and for the outlet, all contained in plastic housing. The dark color of the electrode originates from its use with the carbon-based slurry electrode.

A 0.10 × 0.10 m^2^ cross-flow stack (REDstack B.V., the Netherlands) was used. The design can be found in the literature.^31^ Each end plate had a Ti-mesh 1.0 electrode with 2.5 μm Pt coating to act as a current collector (MAGNETO Special Anodes BV, the Netherlands). Silicon gaskets were used for sealing. The electrode configuration for the flow electrodes was the same as that used for the hexacyanoferrate solutions to make a direct comparison with previous research (Figure 1). Only the end membranes used in this test are AEMs.

**Figure 2:**
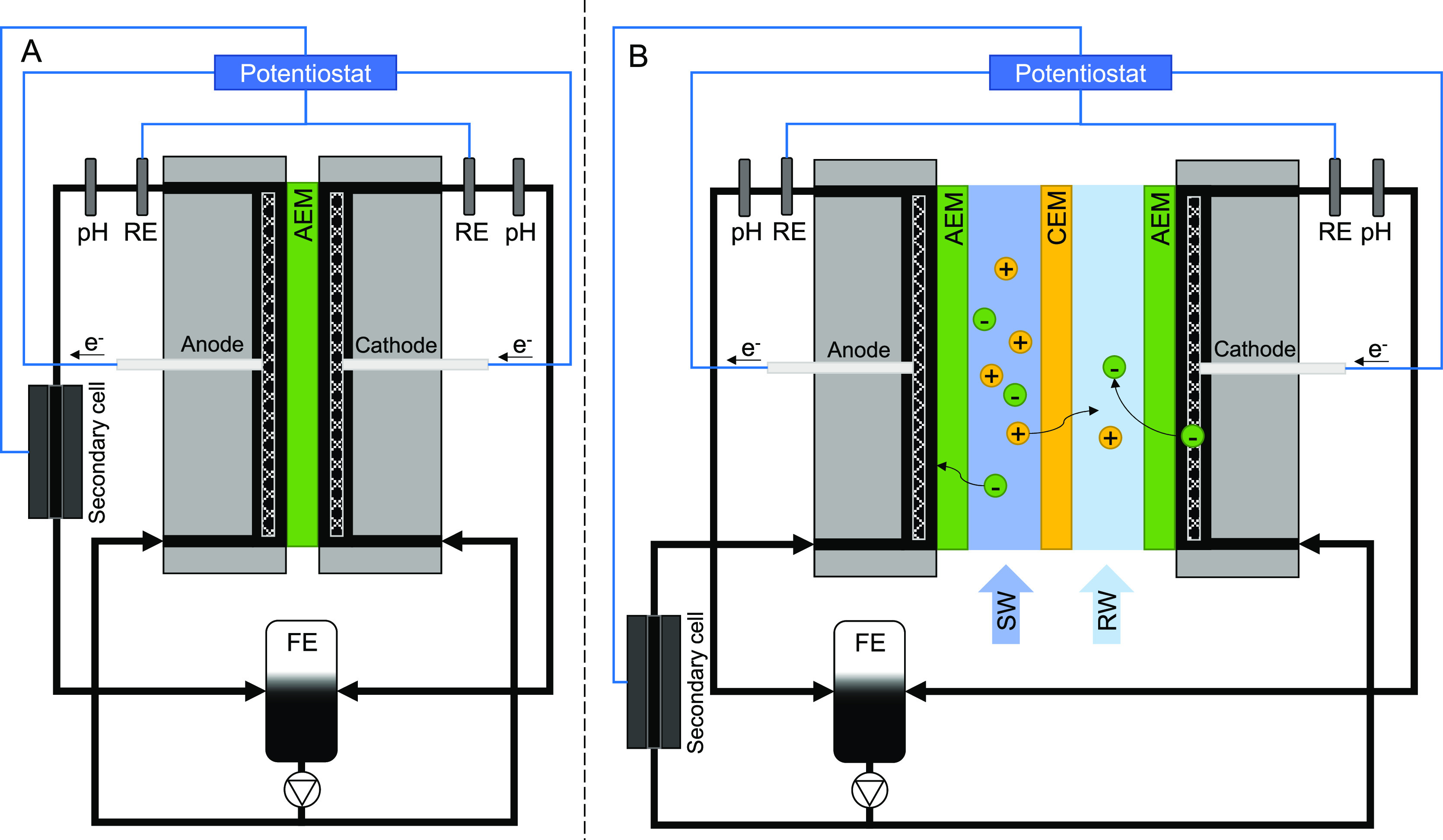
Schematic view of the experimental setup. (A) for single membrane tests and (B) for multiple cell pair tests (one cell pair is shown for simplicity). Carbon particles will become positively charged at the anode and negatively charged at the cathode.

For single-membrane tests, schematized in Figure 2A, one AEM was used (0.01 m^2^ membrane area). If natural waters are supplied, an AEM is needed to block divalent cations to pass from the feedwaters to the flow electrode. While using CTAB, this membrane is also desirable. In laboratory tests, using only NaCl-containing solutions a cation exchange membrane (CEM) can also be used as a shielding membrane or must be used in the case of hexacyanoferrate solutions. For the RED operation (Figure 2B), the stack contained 10 cell pairs, with a total membrane area of 0.20 m^2^. All AEMs and CEMs were Type 10 (FujiFilm Manufacturing Europe B.V., the Netherlands) and were conditioned in 0.5 M NaCl solution for 24 h before being used in the RED stack (characteristics are shown in Table S2). To separate the membranes, 155 μm-thick woven spacers with 55% porosity with integrated silicon sealing were used (Deukum GmbH, Germany).

A secondary electrochemical cell was used in the system to measure inline the CSE resistance using electrochemical impedance spectroscopy (EIS). This cell contained a single flow channel of 1.5 cm thickness and 22 cm^2^ area with two graphite plates that functioned as the cathode and anode (Figure S1).

The CSE was recirculated at 300 mL·min^–1^ using a peristaltic pump with one double pump head (Cole-Palmer, Masterflex L/S Two-Channel Easy-Load II, USA) at the end compartments. Recirculation of the CSE was done in parallel to evaluate the anode and the cathode individually, and the outlets were mixed in the glass bottle. Yet, the anode and cathode can also be connected in series. The slurry was continuously stirred with a propeller stirrer at 600 rpm (CAT, R18, Germany) in the mixing bottle, to promote neutralization of the charged particles and homogeneity. Ag/AgCl reference electrodes (ProSense, the Netherlands) and pH sensors (Digital Orbisint, Endress + Hauser, Germany) were added at the outlet of the anode and the cathode compartments. The absolute pressure was measured with calibrated sensors (MIDAS SW, JUMO GmbH, Germany) at the inlet and outlet of the anode and cathode compartments. Data were collected with a data logger (Memograph M, Endress + Hauser, Germany).

For complete RED operation, artificial seawater and river water with 30.0 and 1.0 g·L^–1^ NaCl (Regenit, Esco, the Netherlands) were pumped into the water compartments at a superficial flow velocity of 1.0 cm·s^–1^ (without accounting for the porosity of the spacer). The feedwater system can be found in previous work.^32^ The outlet flow rates were measured gravimetrically. The temperature and conductivity were measured at the inlet and outlet of each stream (Vstar22, Thermo Fisher Scientific, USA), to quantify the degree of mixing. At the same points, the absolute pressure of each stream was recorded with calibrated sensors (MIDAS SW, JUMO GmbH, Germany). The inlet temperature was set to 25.0 °C (heat losses through the tubing were registered).

### Electrochemical Measurements

First, electrochemical measurements were performed with a single membrane configuration (Figure 2A) to evaluate the anode and cathode potential of the CSE, electrochemical stability and flowability. The open circuit voltage (OCV), the current–voltage (*I*–*V*) curve, the maximum current at 1.15 V, and the stack potential at 50 A·m^–2^ were measured using a potentiostat (IVIUM n-stat, IVIUM Technologies BV, the Netherlands).

The OCV is the stack potential when no current is applied, for the single membrane the cell voltage should be 0 V. The *I*–*V* curve consisted of current density steps of 10 A·m^–2^ for 1 min until 1.2 V was reached. The maximum current density was defined as the current density that could be achieved at 1.15 V cell voltage during a short period of 10 min. This value was chosen to be below the water-splitting voltage of 1.23 V, which is undesirable since it can lead to gas formation. Finally, the slurries were tested with an applied fixed current density of 50 A·m^–2^ for 20 h to evaluate the CSE stability. The value of the current density was chosen to include typical current density values of RED. After the 20 h fixed current density test, the *I*–*V* curve and maximum current density at 1.15 V were repeated.

Second, electrochemical measurements were performed in a 10-cell pair RED stack (Figure 2B), to evaluate the suitability of each CSE with the RED process. The OCV, the *I*–*V* curve, and the performance were measured with a potentiostat. The *I*–*V* curve consisted of current density steps of 5 A·m^–2^ for 2 min until the stack potential reached 0 V. From the *I*–*V* curve, the power density versus current density curve was calculated. The current density at the maximum power density was tested for 1 hour. With the stack voltage as a function of the maximum current density, the gross power output, thermodynamic efficiency, and energy efficiency were calculated. Gross power is the stack voltage output (in this case not corrected for the voltage losses at the electrodes) multiplied by the extracted current. Thermodynamic efficiency is the gross power divided by the mixing energy (per second) expended in the stack (inlet–outlet). Energy efficiency is the gross power divided by the energy (per second) provided at the inlet. Calculations for the mixing energy (Δ*G*) can be found in previous work.^32^ The tests above were done twice for each slurry.

Finally, using CSE 2, electrodialysis experiments were performed for desalination purposes. The same setup was used but now feeding seawater to both water compartments. Different current densities, from 50 to 350 A·m^–2^, were applied to the stack having the stack voltage measured with the potentiostat (IviumStat.h, IVIUM Technologies BV, the Netherlands).

To determine the flow electrode resistance, while the measurements at the main cell took place, electrochemical impedance spectroscopy was applied at the secondary electrochemical cell (Figure S1), with a frequency range from 1 Hz to 250 kHz using another channel of the same multichannel potentiostat.

## Results and Discussion

### Physical Properties of the Carbon-Based Slurry Electrodes

Three types of carbon were selected to prepare the CSEs. AC was the main material used for carbon electrodes for both static and flow electrodes.^16^ CB and GP are known to enhance charge transfer by facilitating the collision between particles and increasing the flow electrode conductivity, compared to pure AC electrodes.^26,33,34^Table 2 shows the BET analysis results of the selected carbons.

**Table 2:** BET Analysis Results for Specific, External, and Internal Surface Area (SA) and Average Pore Size of AC, CB, and GP^a^

carbon type	specific SA [m^2^·g^–1^]	external SA [m^2^·g^–1^]	internal SA [m^2^·g^–1^]	av. pore size [nm]
AC (YP-50F Kuraray)	1665.5	100.2	1565.3	3.6
CB (Monarch 800 Cabot)	233.1	168.3	64.7	21.8^b^
GP (Pure Merck)	11.7	11.2	0.5	10.9

a Figure S2 contains more specific results regarding the BET analysis.

b This value is not consistent with the literature, bigger pore size than the particle size, probably because the pore size detected is between agglomerated particles.

The specific surface area of AC was at least seven times higher than for the CB used and 142 times higher than for the GP used, as seen in Table 2, providing a higher absorption surface. Most of the surface area of AC is internal, while for the other two it is mostly external. The measured value for the CB average pore size was due to the agglomeration of CB particles, which is also seen in the SEM image (Figure S4). CB particles have sizes between 5 and 20 nm;^35,36^ thus a pore size larger than the particle size cannot be correct. However, by agglomeration, the CB particles form primary and secondary structures as detected in SEM imaging and can range up to 10 μm (Figure S4).^35^ A sturdy mixing is necessary to effectively have very finely divided CB particles. For AC and GP, SEM images are also found in the Supporting Information (Figures S3 and S5).

Two physical parameters of special interest in flow electrodes are the slurry viscosity since flowability is key for a good distribution/mixing and the pressure drop at the electrode compartment. A low-pressure drop is required to keep the energetic costs associated with pumping the slurry acceptable.

**Figure 3:**
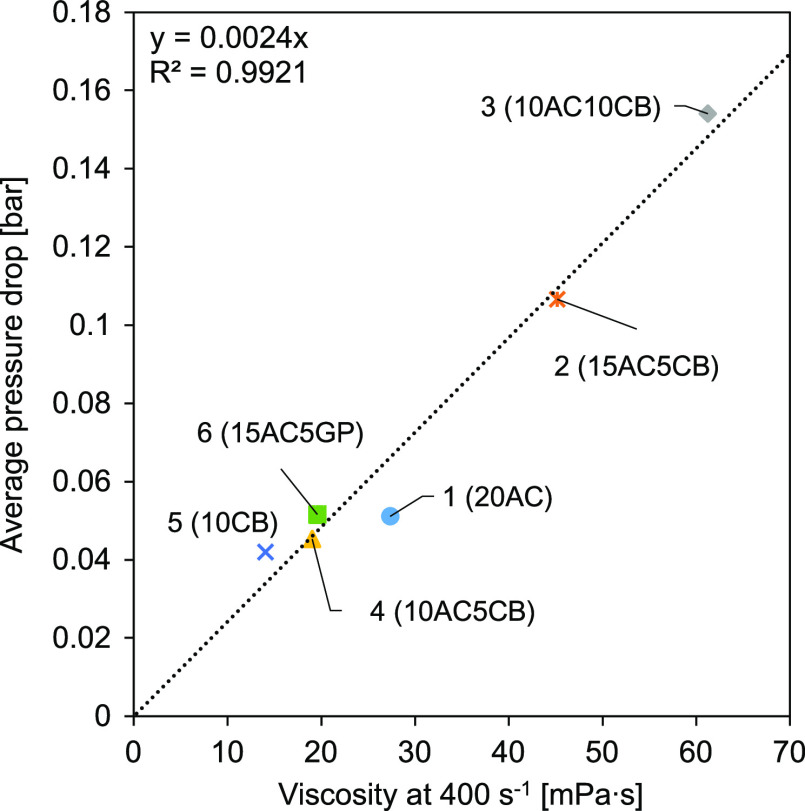
Relation between CSE viscosity (at 400 s^–1^ shear rate) and the average electrode compartment pressure drop. Pressure drop values are based on initial measurements.

Figure 3 shows the relation between the slurry viscosity (at 400 s^–1^ shear rate) and the average pressure drop as measured by flowing through the electrode compartment at 300 mL·min^–1^. A linear relation was found between viscosity and pressure drop as predicted by theory. Figure S6, in the Supporting Information, provides a shear rate range from 50 to 400 s^–1^ for each flow electrode. CSEs are thixotropic fluids (non-Newtonian fluids) or shear-thinning fluids since they become less viscous with agitation/stress.^37^ Looking at each CSE composition more relations were found. Increasing the CB content in the sample (comparing CSEs 1, 2, and 3) also increased the slurry viscosity, while adding GP reduced the viscosity (CSEs 1 and 6). Reducing the carbon weight percentage, as expected,^26^ reduced the viscosity of the CSE. Although CB is much more viscous than AC or GP, the CSE 5 viscosity was lower but still close to the value of CSE 4 and CSE 6. The CSEs that presented lower viscosity and consequently lower pressure drop are more suitable since less energy is spent pumping the CSE. Finally, although a relation was found between viscosity and pressure drop since these are thixotropic fluids, it is relevant to directly measure the pressure drop to estimate the power spent pumping the CSE as the flow rate used will influence the shear rate.

### Performance with a Single Membrane

**Figure 4:**
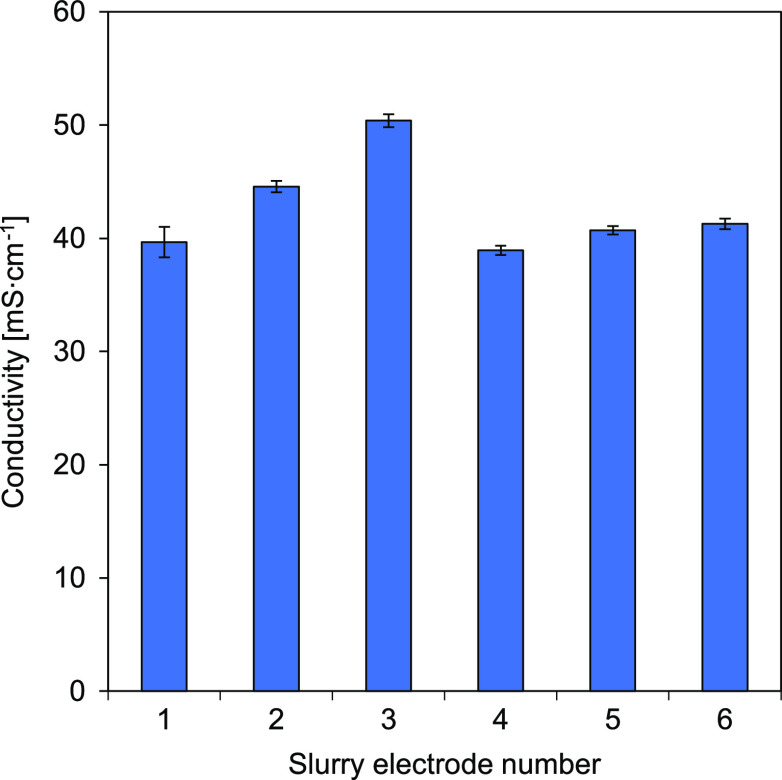
Carbon-based slurry electrode conductivity measured in the secondary cell (Figure S1). CSE 1–20% AC; CSE 2–15% AC + 5% CB; CSE 3–10% AC + 10% CB; CSE 4–10% AC + 5% CB; CSE 5–10% CB and CSE 6–15% AC + 5% GP, all weight percentages and with 0.25 M NaCl in solution.

The CSE performance was further evaluated electrochemically in a single membrane setup (Figure 2A). Using the secondary cell, each CSE electronic conductivity was measured as shown in Figure 4. Higher conductivity leads to lower electrical resistance which is ideal to decrease voltage losses at the electrodes of the RED stack.

In the secondary cell, the conductivity of 0.5 M NaCl was also measured; this resulted in a value of 44.3 ± 1.2 mS·cm^–1^. Estimating for 0.25 M NaCl solution, the conductivity is around 22 ± 2 mS·cm^–1^, as in low-NaCl concentration solutions the relation between conductivity and concentration is rather linear.^32^ Comparing these two values with the obtained in Figure 4, for all CSEs, the addition of carbon clearly enhances the electronic path, leading to conductivity values similar to a 0.5 M NaCl solution.

Since all CSEs contained the same NaCl concentration flowing through the same cell, the differences seen in conductivity in Figure 4 can be attributed directly to the carbon composition of each flow electrode. Increasing the content of CB increases the flow electrode conductivity (comparing CSE 1, CSE 2, and CSE 3). The replacement of AC with CB allowed a percolation threshold at lower weight percentages.^38^ In Figure S7, it is possible to see the electrical conductivity of CB slurries increasing with the increase in weight percentage, from pure H_2_O to 11 wt % CB. The type of CB used, Monarch 800, percolated at 1 wt % with 0.1 mS·cm^–1^ conductivity, showing suitability as an effective electric conductive.^39^ Looking at CSE 1 and CSE 6, the addition of GP, however, did not enhance the conductivity; therefore, the GP used showed not to be a suitable conductive additive for our tests. The mixing of different carbon materials and amounts can lead to different interactions, this was also seen by Cohen et al.^40^ Mixing AC with fluidized bed electrodes enhanced the conductivity of the combined electrode compared to the two materials separated. On the other hand, the combination of carbon nanotubes with the fluidized bed electrodes resulted in a lower conductivity for the combined compared to the carbon nanotubes alone. Using only 10 wt % of CB (CSE 4) resulted in a CSE with the same conductivity as 20 wt % AC (CSE 1). CSE 4 presented the lowest conductivity (38.9 mS·cm^–1^), and adding an extra 5 wt % CB showed a positive effect on the conductivity as for CSE 3 (50.4 mS·cm^–1^). The use of CB enhanced the flow electrode’s electric conductivity, thus decreasing the electrical resistance. The suitable weight percentage will depend on several factors together such as viscosity, conductivity, and dispersibility.^41^

**Figure 5:**
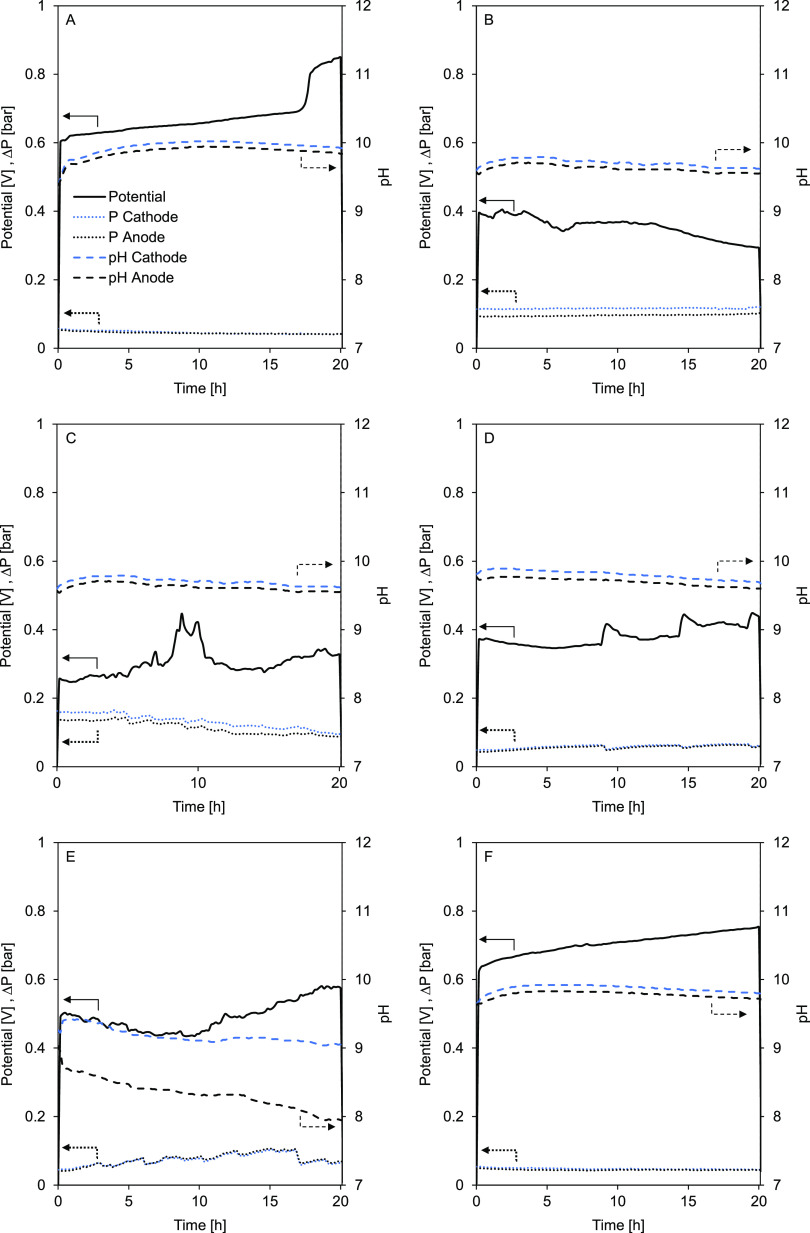
Stability test over 20 h with each carbon-based slurry electrode using a single membrane setup at constant current density (50 A·m^–2^). (A) CSE 1–20% AC; (B) CSE 2–15% AC + 5% CB; (C) CSE 3–10% AC + 10% CB; (D) CSE 4–10% AC + 5% CB; (E) CSE 5–10% CB and (F) CSE 6–15% AC + 5% GP. The left vertical axis represents the cell potential (solid line) and anode and cathode compartment pressure drop (dotted lines), and the right vertical axis represents the pH (dashed line) of the cathode (black) and anode (blue).

To measure the hydrodynamical and electrochemical stability of the flow electrodes, each flow electrode was pumped continuously for 20 h at 50 A·m^–2^ current density. The cell potential, pH of the anolyte and catholyte, and the anode and cathode compartment pressure drop were recorded during this 20 h period and are shown in Figure 5.

In Figure 5, a fluctuation in cell voltage can be seen in all cases. The CSEs that do not contain CB (CSE 1 and 6, Figures 5A,F) showed a linear increase in potential over time (except for the last hours of CSE 1 where a sudden increase was detected). The continuous increase in potential may lead to values above the water electrolysis voltage (∼1.23 V), after a few days of operation if this trend continues. This voltage will trigger oxygen and hydrogen evolution, as well as chlorine gas evolution. This will result in extra potential losses at the electrodes (due to the reactions and gas bubbles formation). Therefore, it is undesired, and these are not considered electrochemically stable. Regarding pH and compartment pressure drop, these two CSEs seem to be stable. In Figure 5B, CSE 2 showed a particular positive behavior by slightly improving over time concerning the decreasing cell potential and also showed a constant pH and pressure drop.

For CSE 3 (Figure 5C), the pressure drop at the electrode compartments decreased over time, as could be caused by the partial settling of the carbon in the tubing, making the flowable fraction less viscous and containing less carbon. This could explain also why the cell potential slightly increases. The pH remained similar in both the anode and cathode compartments, meaning no electrolysis occurred. In Figure 5D, CSE 4 showed peaks in the measured cell potential but also recovered. Interestingly, the potential peaks seem to match the small pressure drop peaks; therefore, it could be an influence of the pump. The pH was also kept similar in this case. Using only CB, with CSE 5 in Figure 5E, it was not possible to avoid side reactions and the pH at the cathode and anode differentiated, indicating water splitting at the current collectors. Furthermore, the potential and pressure drop fluctuated over time.

Figure 5 unveils that by monitoring the flow electrodes for 20 h, the physical and electrochemical characteristics changed. Furthermore, we conclude that flow electrodes containing a mixture of AC and CB offer improved operational conditions with low cell potential and no pH changes.

**Figure 6:**
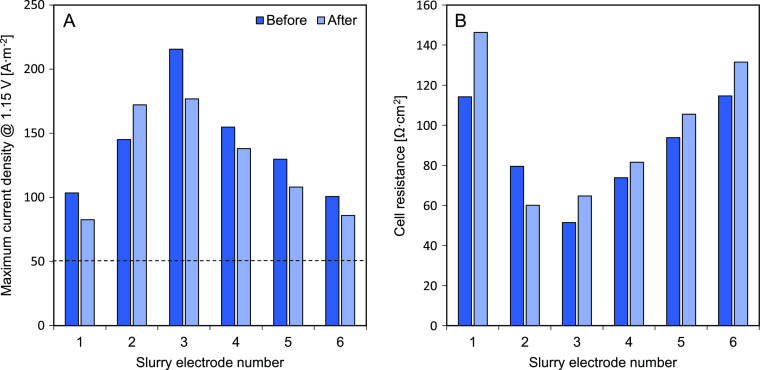
(A) Maximum achieved current density at constant cell potential (1.15 V) and (B) cell resistance taken from the *I*–*V* plot for each carbon-based slurry electrode before and after the 20 h constant current test for a single membrane setup. CSE 1–20% AC; CSE 2–15% AC + 5% CB; CSE 3–10% AC + 10% CB; CSE 4–10% AC + 5% CB; CSE 5–10% CB and CSE 6–15% AC + 5% GP, all weight percentages and with 0.25 M NaCl in solution.

The maximum current density sustained by each flow electrode before reaching the water-splitting voltage and the cell resistance before and after the 20 h testing are shown in Figure 6. Figure 6A reveals that all the tested flow electrodes reached current densities above 50 A·m^–2^ at 1.15 V, which is typically a high current density value suitable for RED. An operational current density above 150 A·m^–2^ is found for CSEs 2 and 3, which also opens the possibility to other applications where higher current densities are needed, such as ED and this is discussed therefore in Section 3.5. Except for CSE 2, the CSE showed decreasing current density over time by up to 20%. This is consistent with the cell electrical resistance increase, seen in Figure 6B.

**Figure 7:**
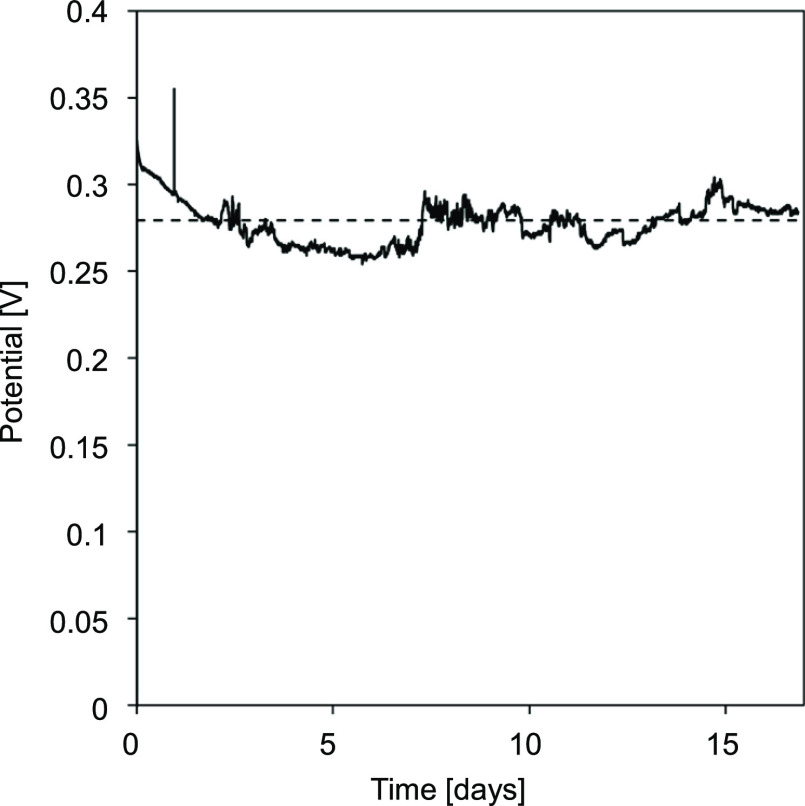
Cell potential measured during a 17 day experiment with a single membrane with CSE 2 (15 wt % AC + 5 wt % CB + 0.25 M NaCl) at 50 A·m^–2^. The potential peak after one day was due to sampling. As sampling disturbed the measuring system and reduced the active volume, no more samples were taken.

Given the fluctuation in cell voltage and cell resistance shown in Figures 5 and 6, CSE 2 was selected for a long-duration test with a single membrane. This CSE was chosen because it was improving over 20 h, being interesting to see if it would stabilize and if so, at what performance level. Figure 7 shows the recorded potential response for a constant current density (of 50 A·m^–2^) through a period of 17 days of a newly made CSE 2.

During the first two days, there was a clear decrease in the cell potential, thus the cell resistance was also decreasing. This was consistent with the results from Figures 5 and 6, probably due to some grinding of the AC/CB mixture leading to finer particles. Figure 7 proves that it is possible to continuously pump the slurry around for at least 17 days (without redispersing with the UltraTurrax) and to maintain a rather stable cell potential. The fluctuation detected initially flattened through time. Although the pressure drop was not measured continuously, there were no signs of clogging during the experiment.

### Performance with a RED Stack

**Figure 8:**
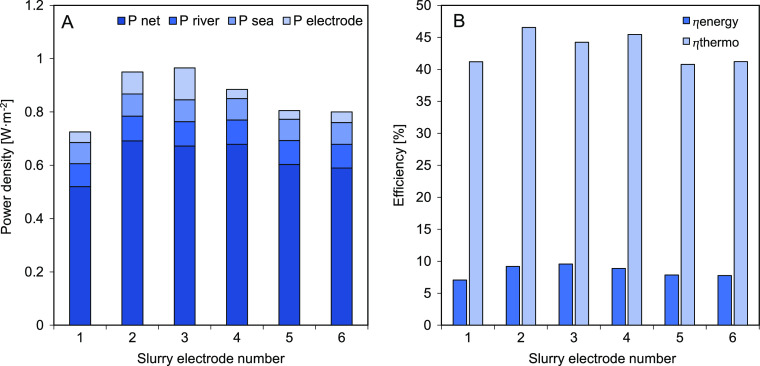
(A) RED performance in terms of gross and net power density, power loss for the river compartment, power loss for the seawater compartment, and power loss for the electrode compartments; (B) energy and thermodynamic efficiency for the tested carbon-based slurry electrodes for a RED stack with 10 cell pairs. 1–20% AC; 2–15% AC + 5% CB; 3–10% AC + 10% CB; 4–10% AC + 5% CB; 5–10% CB and 6–15% AC + 5% GP, all weight percentages and with 0.25 M NaCl in solution.

Since it was shown in the previous section that the CSEs can sustain enough current density for RED, these were tested in an actual RED stack (Figure 2B). Figure 8 shows for each CSE the obtained power density for the RED stack and the corresponding energy and thermodynamic efficiencies. The stack comprised a limited number of 10 cell pairs; therefore, the resistance attributed to the electrode compartments (including the flow electrode) and the extra shielding membrane still contributed significantly to the gross power output, see Figure 8A.

In Figure 8A, the gross power density (aiming for maximum power) is given for the CSEs as used. We found that the power output was stable during the experimental run time. The *I*–*V* and power curves can be found in Figure S8. Since no other condition was changed, apart from the CSE composition, the change in gross power density is due to the different electrical resistance of the CSEs. The obtained results in Figure 8A agree with the single membrane experiments where the slurries with higher electrical resistance in Figure 6B (CSE 1, 5, and 6) showed less gross power density being produced.

Furthermore, Figure 8A categorizes the contributions to the net power density (losses at the river water, the seawater, and the electrode compartments). The power density lost by pumping the sea and river water was constant in all cases; the difference remained in the flow electrode pumping power density contribution which varied according to the viscosity of the CSE. This led to a very similar net power density of 0.69, 0.67, and 0.68 W·m^–2^ for CSEs 2, 3, and 4, respectively. Nonetheless, it must be emphasized that the number of cell pairs (10) is small, and therefore for a larger number of cell pairs, both the pumping power losses at the electrodes and the electrode resistance will be negligible compared to the pumping power loss at the water compartments and total cell pair resistance.^10^ Thereby, according to this test, all CSEs, except CSE 5 due to the change in pH under current, could still be suitable for RED.

The energy efficiencies obtained, in Figure 8B, were for all cases between 7 and 10%. This is common for small stacks and can be mostly attributed to the short residence time of 10 s.^2,42^ Not correcting the power density for the voltage losses at the electrodes given only 10 cell pairs also lowers the energy efficiency. Longer residence times would increase the energy efficiency but reduce the power density.^42^ Other known strategies that can increase the efficiency are electrode segmentation^32^ or multistage^43^ without sacrificing the power density and can also be implemented with carbon-based slurry electrodes. The thermodynamic efficiencies were above 40% in all cases and were close to the theoretical maximum of 50% while aiming also for maximum power,^44^ which was the case in these experiments.

### Comparison to Other Alternatives

To further determine the suitability of the best slurries presented in this study (CSE 2, CSE 3, and CSE 4), Table S3 compares and evaluates these CSEs with other known electrode systems for RED. The stack properties and water residence time influence the gross power density; therefore, these parameters are first described. Electrode rinse solutions, such as iron chloride, have a technological potential like hexacyanoferrate solutions; therefore, these are not specified in this evaluation. An extensive evaluation of suitable electrode systems for RED has been reported by Veerman et al.^3^ For comparison, the following parameters are used: gross power density, water switching needed (intermittency), and technological potential. The technological potential includes sustainability, safety, scalability, economical sustainability (e.g., materials costs), and performance (e.g., gross power density), as assessed by us. More details can be found in Tables S3 and S4.

From analyzing Table S3, it is concluded that the most promising electrode systems are the slurries with AC and CB, in particular, CSE 2 and CSE 3 because they provide the best properties in terms of good electrochemical and physical performance, sustainability and safety, scalability, low pressure losses at the electrode compartment and no need to switch the river water and seawater flow contrary to CRED, and the low cost of the carbon materials (Table S4). The gross power density results with NaCl, CRED,^10^ or F-CAPMIX^45^ are more than 3 times lower than the obtained results with CSEs. This may indicate that the electrode compartment voltage losses are higher in these systems. Furthermore, CRED and F-CAPMIX have the disadvantage of being intermittent. The hexacyanoferrate system provides the highest gross power density; however, due to stability and environmental concerns, it is not a suitable option.

The pumping losses for the CSE are influenced by the electrode compartment geometry. Recent studies revealed serpentine flow field geometries for the electrode caused pressure losses such high that net power density values even became negative.^46^ In this study, the pressure drop loss was negligible for the flow electrodes, even though the electrode compartments were not further adapted for a slurry. This may still be improved in a dedicated study, also aiming to scale up to larger stacks.

The use of a surfactant was studied as an option to stabilize the slurries. In this case, CTAB was used, aiming to improve the CSE’s dispersibility and avoid settling of the mixed carbon slurry (if necessary). However, the addition of such a surfactant was found to affect negatively the CSE electrochemical performance as the overpotential increased over time. The addition of 7.8 wt % CTAB significantly improved the stability of the mixed CB–AC slurry. However, the use of such surfactants will result in safety and sustainability issues as redox solutions and should be avoided.

Toward a full economic optimization of the electrode compartment, replacing the current collector from Ti/Pt mesh with graphite plates would drastically decrease the costs associated with the electrode compartment, as well as contribute to a more sustainable process by reducing the use of scarce materials like platinum and reducing the chance of water electrolysis which is catalyzed by platinum by taking away the catalytic action of platinum toward water splitting into oxygen and hydrogen. During this research, the use of graphite plates was attempted. However, the current collector modification toward a graphite insert plate while keeping the rest of the RED stack structure intact was not yet successful. This was due to the change of a mesh electrode for a flat plate electrode resulting in a flow path with a poor mixing degree and lack of support for the membrane pile. More work in this area is recommended.

### ED Experiment

**Figure 9:**
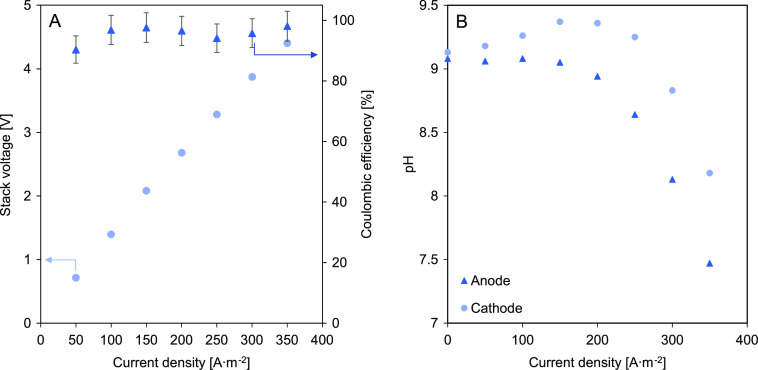
(A) Stack voltage and Coulombic efficiency for ED mode at different current densities using CSE 2. (B) Anode and cathode pH change with current density applied.

The relatively high current densities obtained in the single membrane test allow and stimulate the CSEs to be used in other applications besides flow capacitive deionization, F-CAPMIX, or RED. Therefore, using CSE 2, a short test using the same cross-flow RED stack was performed under ED conditions, feeding seawater at the inlets. Figure 9 shows the stack voltage, Coulombic efficiency, and pH change at different current densities.

The measured stack voltage was linear with the applied current density, and no limiting voltage was detected. The Coulombic efficiency achieved values of 90% in all cases. This value might be overestimated due to the conversion between conductivity (mS·cm^–1^) to concentration (mol·L^–1^), while not considering the water transport as the outlet flow rate was not measured. The Coulombic efficiency was calculated according to Doornbusch et al.^47^ However, the pH measurements at the anode and cathode showed that at current densities above 150 A·m^–2^, electrolysis occurred with the anode becoming acidic and the cathode becoming alkaline. The reason for the drop in pH at the cathode above 200 A·m^–2^ is not known. The application of CSEs for electrodialysis is advantageous for a continuous process without redox reactions, particularly in specific configurations in which waters cannot be reversed due to, for example, the use of bipolar membranes or the need to keep the solution in a determined compartment. The results show potential application; nonetheless, a dedicated study on carbon-based slurry electrodes for ED is advised.

## Conclusions

CSEs were tested to move toward redox-free reverse electrodialysis as these slurry electrodes allow a continuous RED process in a more clean and sustainable way. Several compositions of CSEs were tested including mixing AC with CB or GP as conductive additives. The CSEs were characterized both physically and electrochemically. From these tests, the CSEs that performed best comprised a mixture of AC and CB (CSE 2, CSE 3, and CSE 4) presenting low electrode losses and stable electrical performance. However, in the case of a higher loading of carbon black (10 wt %, CSE 3), the viscosity increased considerably, thereby increasing pumping losses for the electrode compartments. By achieving current densities higher than 150 A·m^–2^ with CSE 2, CSEs can also be used for desalination with ED. It is recommended to further test these CSEs to evaluate the effect of multivalent ions present in the seawater and river water and validate the long-term operational stability.

## Supporting Information Available

Details for carbon-based slurry preparation (Table S1), ion exchange membranes characteristics (Table S2), schematic of secondary cell used to perform EIS measurements (Figure S1), details of the BET analysis of the carbon materials (Figure S2), SEM images of the carbon materials (Figures S3–S5), viscosity measurements of the carbon-based slurry electrodes (Figure S6), electrical conductivity of carbon black (Figure S7), *I*–*V* and power curves for each carbon-based slurry electrode (Figure S8), and comparison with alternative electrode systems (Tables S3 and S4) (PDF)
