# Supplementary material for: Toward Redox-Free Reverse Electrodialysis with Carbon-Based Slurry Electrodes
Source: Ind Eng Chem Res. 2023 Jan 14;62(3):1665–75. doi: 10.1021/acs.iecr.2c03567 (PMC9881007; doi:10.1021/acs.iecr.2c03567)
Supplement: Supplementary file 1 — ie2c03567_si_001.pdf [file ie2c03567_si_001.pdf]

Supporting Information

Towards Redox Free Reverse Electrodialysis with Carbon-based Slurry Electrodes

Catarina Simões,[a,b,*] Michel Saakes,[a] Derk Brilman,[b]

[a] Wetsus, European Centre of Excellence for Sustainable Water Technology, P.O. Box 1113, 8900 CC, Leeuwarden, the Netherlands

[b] Sustainable Process Technology, Faculty of Science and Technology, University of Twente, P.O. Box 217, 7500 AE, Enschede, the Netherlands

* Corresponding Author

Catarina Simões

Email: Catarina.simoes@wetsus.nl

Postal address: Wetsus, European Centre of Excellence for Sustainable Water Technology, P.O. Box 1113, 8900 CC, Leeuwarden, the Netherlands

Submitted for publication in Industrial & Engineering Chemistry Research

Summary

Number of pages: 8, Figures: 8, Tables: 4.

Slurry preparation

For each carbon-based slurry electrode, 700 mL of deionized water were used. First, a third of the water was poured in a beaker. Then the carbons were weighted and placed in the same beaker. Slowly the rest of the water was poured into the beaker. Finally, the NaCl was weighted and added to the mixture. The slurry was mixed manually, using a spoon to avoid the powders to spread. Once the powders were mixture, the slurry was firmly mixed during 12 minutes using an UltraTurrax (IKA, T25, Germany) at 12000 rpm. After preparation a sample was taken for viscosity measurements and the remaining slurry was tested first in the single membrane configuration and later in the RED stack.

Table S1 – CSEs composition used for the experiments.

Slurry	Composition (g)					
	H_2O	NaCl	AC	CB	GP	Total
CSE 1 – 20AC	700.00	10.21	177.55	0.00	0.00	887.76
CSE 2 – 15 AC + 5 CB	700.00	10.21	133.16	44.39	0.00	887.76
CSE 3 – 10 AC + 10 CB	700.00	10.21	88.78	88.78	0.00	887.76
CSE 4 – 10 AC + 5 CB	700.00	10.28	83.56	41.78	0.00	835.62
CSE 5 – 10 CB	700.00	10.26	0.00	78.92	0.00	789.18
CSE 6 – 15 AC + 5 GP	700.00	10.21	133.16	0.00	44.39	887.76

Ion exchange membranes characteristics

Table S2 - Characteristics of the ion exchange membranes. Information provided by the manufacturer.

Membrane	Manufacturer	Dry thickness [μm]	Electrical resistance [Ω·cm^2]	Perm selectivity [%]
Type 10 AEM	FujiFilm	125	1.7	95
Type 10 CEM	FujiFilm	135	2.0	99

Secondary cell to perform EIS (Electrochemical Impedance Spectroscopy)

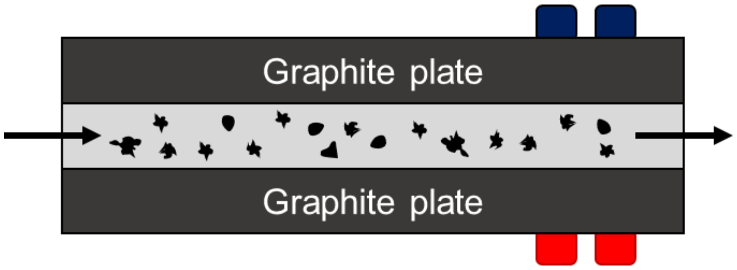

Figure S1 - One chamber electrochemical cell used for EIS

Brunauer-Emmett-Teller (BET) analysis

Samples were degassed under a nitrogen atmosphere for 2 hours at 300 °C in a degassing apparatus (VacPrep 061, Micromeritics, Norcross, GA, USA). The degassing was needed to remove adsorbed gases and water from the pores of the sample material. Subsequently, nitrogen gas adsorption (TriStar 3000, Micromeritics, Norcross, GA, USA) at -196 °C (77 K) was used to determine the specific surface area of the samples according to the BET model.

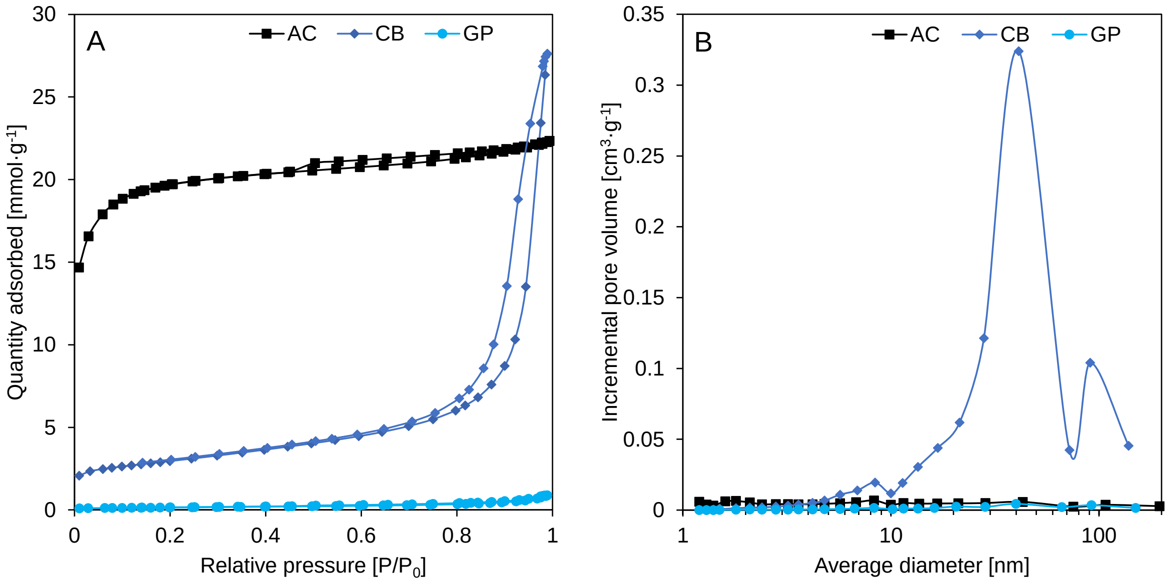

Figure S2 - (A) Absorption and desorption rate of each carbon and (B) incremental pore volume.

Scanning electron microscopy (SEM) of raw carbons

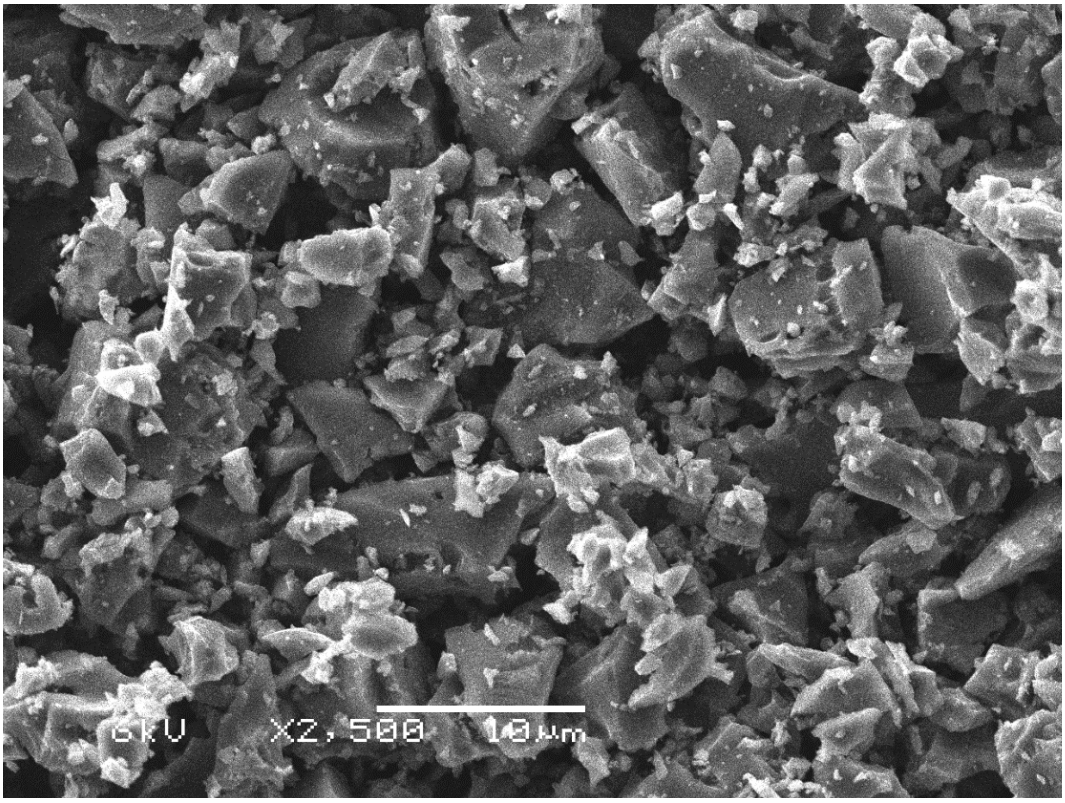

Figure S3 - Activated Carbon (x2500)

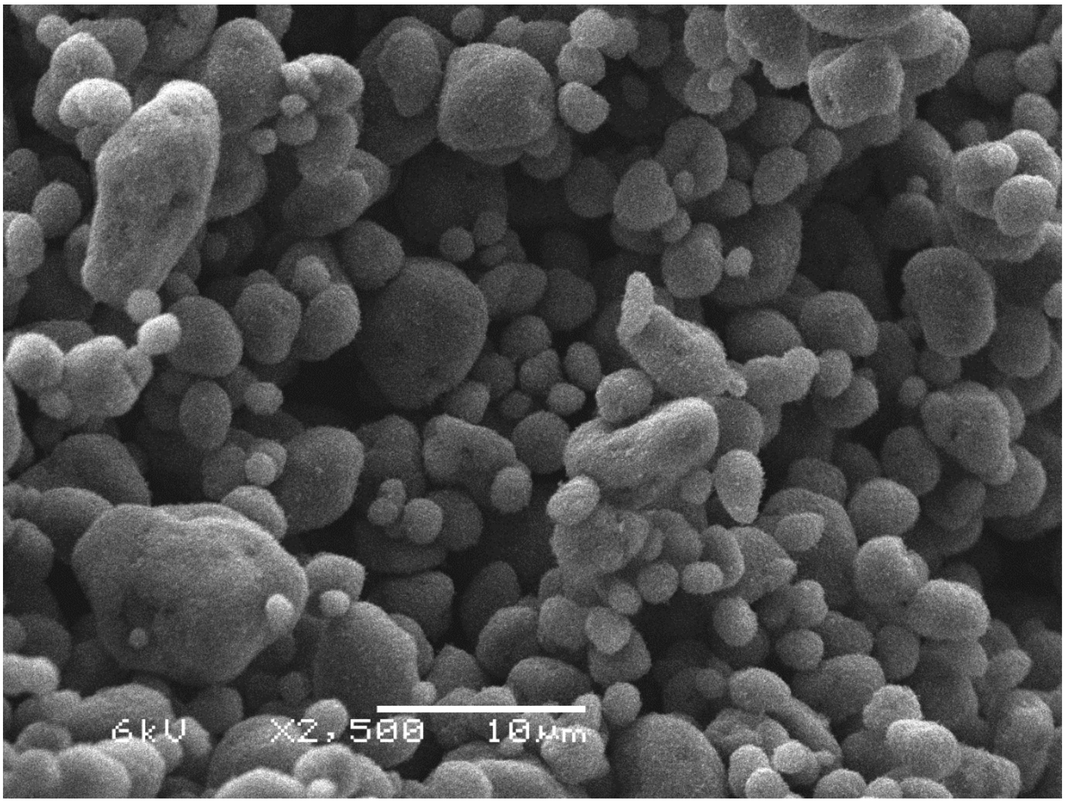

Figure S4 - Carbon black agglomerates (x2500)

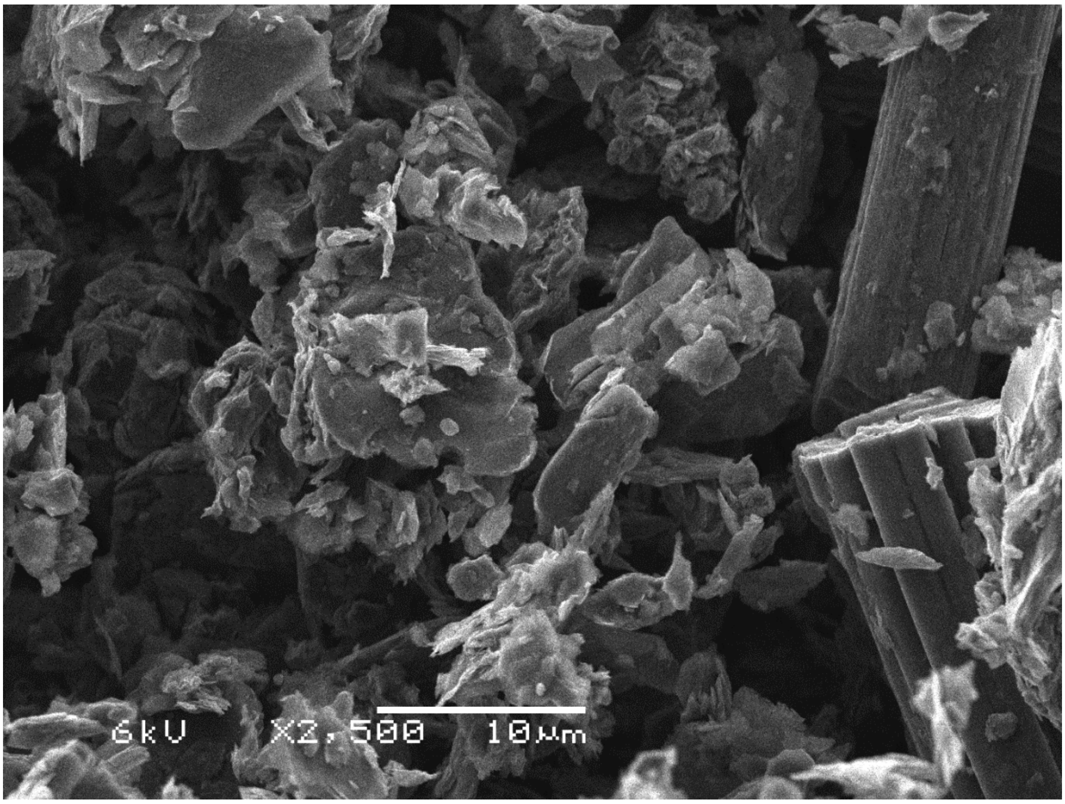

Figure S5 - Graphite powder (x2500)

Viscosity measurements

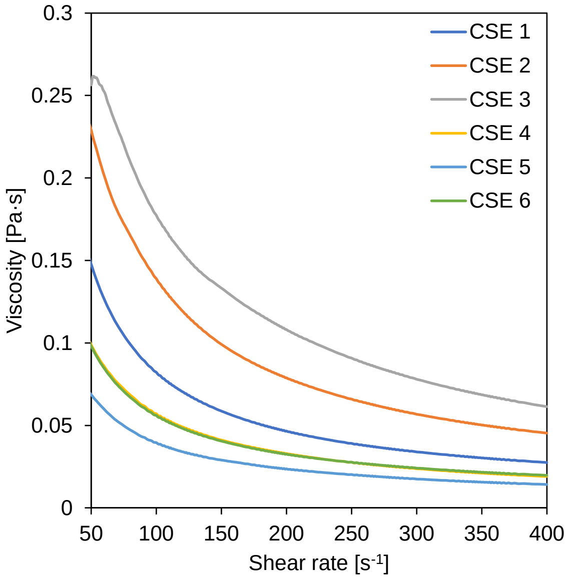

Figure S6 – Carbon-based slurry electrodes viscosity at shear rates from 50 to 400 s^{-1}.

Carbon black electrical conductivity

The test of the carbon black (CB) electrical conductivity was conducted by pumping the solution through the secondary cell only at 300 mL·min^{-1}. The solution initially consisted of one litre of pure H_2O and every three measurements 1wt% of CB was added until 11 wt% was achieved. After 0.25 M NaCl was added to the sample to quantify the salt effect, as shown in position 12, Fig, S7, the carbon slurry composition was 11 wt% CB + 0.25 M NaCl instead of 12 wt% CB.

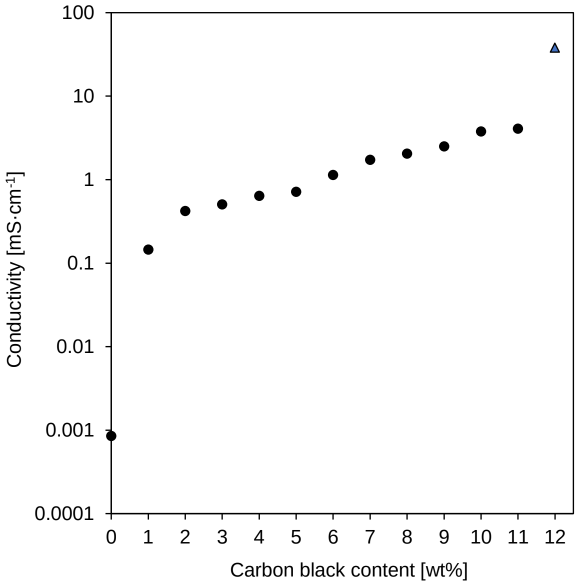

Figure S7 – Electrical conductivity of different carbon black weight percentages in pure H_2O. The last point (12 wt%) relates to a composition with 11 wt% CB + 0.25 M NaCl.

I-V and Power curve for each carbon-based slurry electrode in a 10-cell pair REDstack

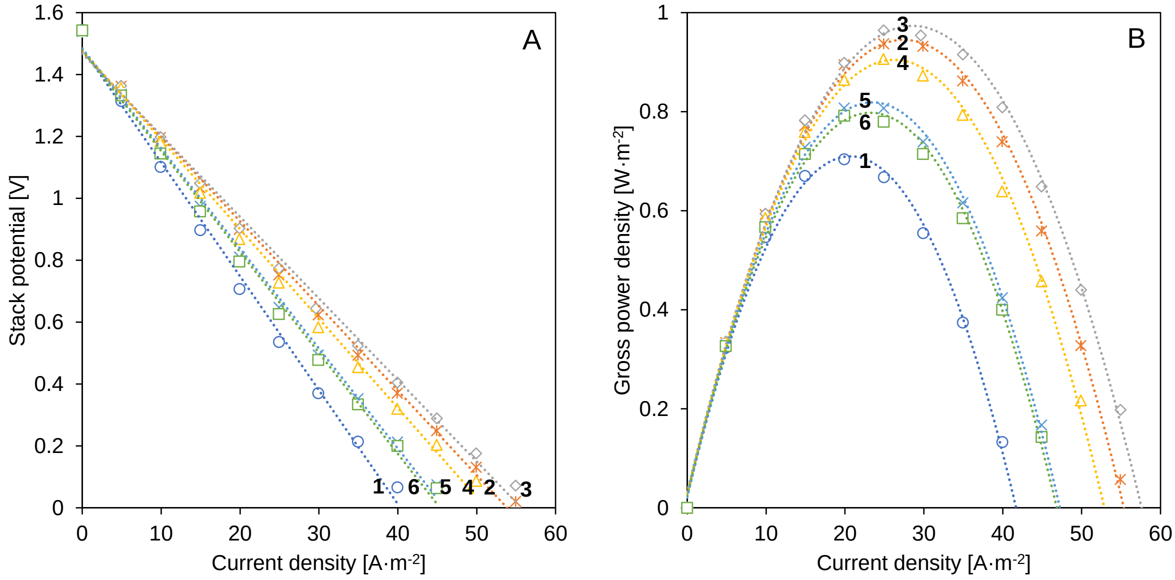

Figure S8 – (A) I-V and (B) power curve changing carbon-based slurry electrode at the electrode compartment for a RED stack with 10 cell pairs. 1 – 20 % AC; 2 – 15 % AC + 5 % CB; 3 – 10 % AC + 10 % CB; 4 – 10 % AC + 5 % CB; 5 – 10 % CB and 6 – 15 % AC + 5 % GP, all weight percentages and with 0.25 M NaCl in solution.

Comparison with alternative electrode systems

Table S3 – Comparison and evaluation of different electrode systems for conventional RED (with $[Fe(CN)_6]^{3-/4-}$ or NaCl), CRED, F-CAPMIX and CSE-RED.

Electrode compartment	Stack details	Residence time [s]	Cell pair number	Flow rate Electrode rinse solution [mL/min]	Gross power density [W·m^{-2}]	Intermittent use of seawater and river water	Technological Potential*	Reference
0.25M NaCl	0.1 x 0.1 m^2 200 µm spacer Neosepta CMX/AMX	10	30	200	0.230	No	+/-	[1]
CRED (90 wt% AC + 10 wt% PVDF)		6 #	10	200	0.760	Yes	+/-	[1]
FE-RED (10 wt% AC + 0.5M NaCl + brushes)	0.0007 m^2 250 µm spacer Neosepta CMX/AMX	n.d.	4	20	0.260	No	+	[2]
F-CAPMIX (5 wt % AC + 0.6M NaCl)	0.00138 m^2 360 µm spacer Neosepta CMX/AMX	n.d.	n.a.	25	0.175	Yes	-	[3]
0.2M $K_3Fe(CN)_6$ 0.2M $K_4Fe(CN)_6$ 0.25M NaCl	0.1 x 0.1 m^2 155 µm spacer Type 10 Fujifilm	10	10	150	1.220	No	-	This study
CSE 2 (15 wt% AC + 5 wt% CB + 0.25M NaCl)		10	10	300	0.945	No	+	This study
CSE 3 (10 wt% AC + 10 wt% CB + 0.25 M NaCl)		10	10	300	0.973	No	+/-	This study
CSE 4 (10 wt% AC + 5 wt% CB + 0.25 M NaCl)		10	10	300	0.905	No	+	This study
CSE 2 with surfactant		10	10	300	0.800	No	-	This study

* Technological potential evaluated by the authors in terms of sustainability and safety, feasibility, economics and performance (-, +/-, +).
\# Accounting with spacer porosity
RED tests used 1 and 30 g NaCl·L^{-1} for sea and river water, respectively. F-CAPMIX used either 35 g NaCl·L^{-1} or deionized water in the central compartment.
n.d. – not defined; n.a. – not applicable

Table S4 – Cost and hazards of the materials used for the electrode system.

Materials	Costs [€/kg]	Hazards at the electrode comp.	References
$K_3Fe(CN)_6/K_4Fe(CN)_6$	~ 400	Cyanide formation, scaling, harmful to the aquatic environment	[4,5]
NaCl	~ 0.7	Cl2 and H2 evolution, scaling	[6]
PVDF	~ 1840	None	[7]
Activated Carbon	~ 10	None	[8]
Carbon Black	~ 3	None	[9]
CTAB	~ 270	Very toxic for the aquatic environment, corrosive	[10]

References

(1) Vermaas, D. A.; Bajracharya, S.; Sales, B. B.; Saakes, M.; Hamelers, B.; Nijmeijer, K. Clean Energy Generation Using Capacitive Electrodes in Reverse Electrodialysis. Energy Environ Sci 2013, 6, 643. https://doi.org/10.1039/c2ee23562e.

(2) Liu, F.; Coronell, O.; Call, D. F. Electricity Generation Using Continuously Recirculated Flow Electrodes in Reverse Electrodialysis. J Power Sources 2017, 355, 206–210. https://doi.org/10.1016/j.jpowsour.2017.04.061.

(3) Kim, H.; Choi, J.; Jeong, N.; Im, H. J.; Yeo, J. G.; Jeon, S. il; Chun, W. G.; Kim, D. K.; Yang, S. Electrochemical Analysis of High-Performance Flow-Electrode Capacitive Mixing (F-CapMix) under Different Operating Conditions. ACS Sustain Chem Eng 2021, 9 (28), 9199–9208. https://doi.org/10.1021/acssuschemeng.1c00848.

(4) Potassium hexacyanoferrate(III), https://www.sigmaaldrich.com/NL/en/product/mm/104973, consulted on 26.08.2022

(5) Potassium hexacyanoferrate(II) trihydrate, https://www.sigmaaldrich.com/NL/en/product/mm/104984, consulted on 26.08.2022

(6) ESCO tableted salt REGENIT 25kg, https://sanresurs.lv/en/esco-sals-tabletes-filtriem-regenit-25kg-(vacija), consulted on 26.08.2022

(7) Poly(vinylidene fluoride), https://www.sigmaaldrich.com/NL/en/product/aldrich/182702, consulted on 26.08.2022

(8) Lithium Ion Battery Conductive Powder YP50F YP80F Active Carbon Super Capacitor Activated Carbon With High Quality, https://www.alibaba.com/product-detail/Lithium-Ion-Battery-Conductive-Powder-YP50F_1600275023235.html?spm=a2700.details.0.0.61e137b6RuGzcn, consulted on 30.08.2022

(9) Black Pearl 800 Cabot Carbon Black, https://carbonblack.en.made-in-china.com/product/SKLmYliJugRT/China-Black-Pearl-800-Cabot-Carbon-Black.html, consulted on 30.08.2022

(10) Hexadecyltrimethylammonium bromide, https://www.sigmaaldrich.com/NL/en/product/sigma/h5882, consulted on 26.08.2022
